# The genomic basis for the evolution of a novel form of cellular reproduction in the bacterium *Epulopiscium*

**DOI:** 10.1186/1471-2164-13-265

**Published:** 2012-06-21

**Authors:** David A Miller, Garret Suen, Kendall D Clements, Esther R Angert

**Affiliations:** 1 Department of Microbiology, Cornell University, Ithaca, NY, 14853, USA; 2 Department of Bacteriology, University of Wisconsin-Madison, Madison, WI, 53706, USA; 3 School of Biological Sciences, University of Auckland, Auckland, 1142, New Zealand

**Keywords:** Developmental process, Endospore, Sporulation, Spore, Endospore-forming bacteria, Binary fission, Intracellular offspring

## Abstract

**Background:**

*Epulopiscium* sp. type B, a large intestinal bacterial symbiont of the surgeonfish *Naso tonganus*, does not reproduce by binary fission. Instead, it forms multiple intracellular offspring using a process with morphological features similar to the survival strategy of endospore formation in other Firmicutes. We hypothesize that intracellular offspring formation in *Epulopiscium* evolved from endospore formation and these two developmental programs share molecular mechanisms that are responsible for the observed morphological similarities.

**Results:**

To test this, we sequenced the genome of *Epulopiscium* sp. type B to draft quality. Comparative analysis with the complete genome of its close, endospore-forming relative, *Cellulosilyticum lentocellum*, identified homologs of well-known sporulation genes characterized in *Bacillus subtilis*. Of the 147 highly conserved *B. subtilis* sporulation genes used in this analysis, we found 57 homologs in the *Epulopiscium* genome and 87 homologs in the *C. lentocellum* genome.

**Conclusions:**

Genes coding for components of the central regulatory network which govern the expression of forespore and mother-cell-specific sporulation genes and the machinery used for engulfment appear best conserved. Low conservation of genes expressed late in endospore formation, particularly those that confer resistance properties and encode germinant receptors, suggest that *Epulopiscium* has lost the ability to form a mature spore. Our findings provide a framework for understanding the evolution of a novel form of cellular reproduction.

## Background

Endospore formation is an ancient and complex developmental process exclusive to certain bacteria within the Firmicutes [1,2]. Endospores endure environmental conditions that would kill most other bacterial cells, including prolonged periods of insufficient nutrients, moderate levels of organic solvents, exposure to phage, extremes in pH, proteases and cell wall degrading enzymes, freezing, desiccation and excessive heat or radiation [3,4]. This form of sporulation preserves the genome in a remarkably dispersible and dormant cell type that can resume vegetative growth when the environment improves. While most sporulating species of Firmicutes produce a single endospore, some have the ability to produce multiple endospores [5]. For example, *Clostridium oceanicum* regularly forms two endospores, one at each end of the mother cell [6]. Others include the Segmented Filamentous Bacteria, a group of uncultivated inhabitants of the intestinal tract of animals [7]. These multicellular filaments live attached to the lining of the small intestine, and to disperse or reposition itself in the gut, each cell in a filament forms either an endospore containing two cells or two non-dormant intracellular offspring [7,8].

Other lineages within the Firmicutes use multiple endospore formation as a reproductive strategy. The guinea pig intestinal symbiont *Metabacterium polyspora* may undergo binary fission but the regular formation of multiple endospores, up to nine from a single mother cell, is a significant form of reproduction [9,10]. The life history of *M. polyspora* may be selecting for this unusual mode of reproduction, which could improve survival as the bacteria cycle in and out of the host gastrointestinal tract [10]. A large and diverse group of surgeonfish intestinal symbionts related to *M. polyspora* display an array of reproductive modes that involve binary fission and/or sporulation [11]. Like *M. polyspora*, the identified morphotypes and phylotypes of these surgeonfish symbionts show host-specific distributions, and an individual fish acquires the symbionts by the ingestion of feces or detritus [11,12]. The type C *Epulopiscium*-like fish intestinal symbionts, rely solely on the formation of two endospores for reproduction and appear to have abandoned binary fission altogether [12]. The largest members of this group of symbionts, *Epulopiscium* spp. type A and type B, are phylogenetically distinct from the smaller endospore-forming C morphotypes. *Epulopiscium* spp. type A and B appear to have taken the developmental process one step further and reproduce by the daily production of two or more intracellular offspring that are not dormant [11,13]. The phylogenetic relationship between multiple endospore formers and lineages that produce non-dormant intracellular offspring, and the morphological changes shared between these processes, suggest that the latter developmental process is related to endospore formation [13].

**Figure 1:**
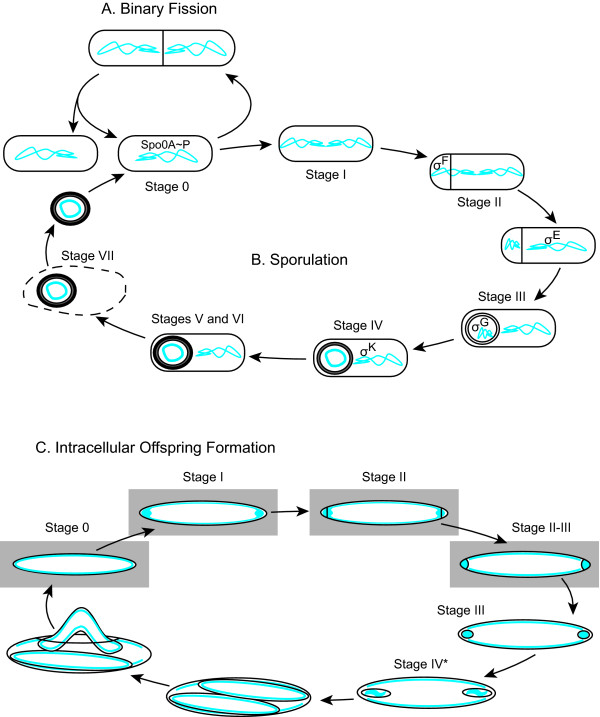
**The life cycles of *B. subtilis* and *Epulopiscium* sp. type B. A**) In a favorable environment, *B. subtilis* undergoes growth and division. **B**) When nutrient limitations become critical, the cell may develop an endospore. Shown here are the morphological stages described for sporulation. The temporal and spatial activation of Spo0A and the four sporulation-specific sigma factors are shown. The grey circle around the forespore indicates the cortex. The thick black circle around the forespore at stage V and beyond represents the spore coat. **C**) Earliest stages of offspring development in *Epulopiscium* sp. type B are based on the similar morphological transitions described for sporulation in *B. subtilis*. See text for a detailed explanation of the process. Offspring frequently initiate the next round of reproduction prior to exiting the mother cell. Those stages that are seen in offspring still within their mother cell are highlighted with grey boxes. For all diagrams, DNA is shown in blue.

The stages of endospore formation (Figure 1) are described in the model organism *Bacillus subtilis*[14,15] and these morphological transitions appear conserved in other endospore formers [16]. Cells that exhibit no overt signs of sporulation are defined as stage 0. After initiation of sporulation, the chromosome replicates and replication origins become tethered to opposite poles of the cell. This unusual nucleoid conformation is called the axial filament and these cells are said to be in stage I [17]. Instead of dividing at the midcell, the sporulating cell divides near one pole, producing the forespore and larger mother cell, which marks stage II. Division traps approximately one-third of one of the chromosomes in the forespore [18]. The rest of the chromosome, still within the mother cell, is translocated into the forespore so that the spore contains a complete genome [19]. Enzymatic degradation of peptidoglycan between the mother cell and forespore results in curvature of the septum [20,21]. The mother-cell membrane then wraps around the forespore to completely engulf the forespore, which marks stage III [22]. In stage IV, a modified peptidoglycan called the cortex is synthesized in the space between the mother-cell and forespore membranes. In stage V, a complex proteinaceous coat is applied to the developing spore [23,24]. Stage VI is defined as endospore maturation, when the spore gains many of its resistance traits [3]. Lastly, the mother cell lyses to release the mature spore, in stage VII.

The sequential activation of stage-specific transcription factors controls the proper timing and location of gene expression to ensure the progression of cell-specific developmental events. In *B. subtilis*, a network of kinases and phosphatases conveys information about intracellular and extracellular conditions to the phosphorelay, which ultimately determines the phosphorylation state of the transcription factor Spo0A [25-27]. While Spo0A is considered the master regulator of sporulation, it also regulates a number of alternative cellular reactions to environmental change [28]. In its active form, Spo0A ~ P either directly or indirectly affects the transcription of more than 500 genes [29]. Spo0A activation is essential for entry into sporulation.

After asymmetric division, gene expression is regulated by four sporulation-specific sigma factors: σ^F^, σ^E^, σ^G^, and σ^K^ (Figure 1) [30,31]. σ^F^ and σ^G^ are activated only in the forespore while σ^E^ and σ^K^ are activated only in the mother cell. Both σ^F^ and σ^E^ are expressed prior to asymmetric division [32], but σ^F^ is held inactive in a complex with two peptides of its anti-sigma factor SpoIIAB [33] and σ^E^ is synthesized as an inactive pro-peptide [34]. The forespore sigma, σ^F^, is the first to be activated. SpoIIE phosphorylates SpoIIAA (the σ^F^ anti-anti-sigma factor), which binds SpoIIAB leading to the release of σ^F^[35-37]. SpoIIR, part of the σ^F^ regulon [38], is produced in the forespore and inserted into the sporulation septum, where it activates SpoIIGA in the mother cell [39]. SpoIIGA then cleaves pro-σ^E^ thus releasing mature σ^E^ to the cytoplasm [40]. Likewise, the late-sporulation sigma factors, σ^G^ and σ^K^, are not immediately functional when expressed and activation of each entails factor-specific intracellular signaling cascades and release mechanisms [41-48].

Tighter control of particular genes in each regulon is provided by additional transcription factors [49-53] that form both coherent and incoherent feed-forward loops with their associated sigma factor [1]. Coherent feed-forward loops occur when a sigma factor regulates expression of a gene and then combines with that gene product to up-regulate more genes. Incoherent feed-forward loops come about when such a combination leads to the down-regulation of additional genes [1,53]. For example, *rsfA* and *spoIIR* are both expressed from σ^F^ promoters [38,49]. However, RsfA combined with σ^F^ turns off transcription of *spoIIR*, so only a brief burst of *spoIIR* expression is seen immediately following asymmetric division. Such feed-forward loops allow the cell to modulate the timing, duration and location of expression of subsets of genes within a regulon. The combination of all central transcriptional regulatory mechanisms modulates the expression of more than 700 genes during sporulation.

The *B. subtilis* model can serve as a foundation for exploring mechanistic modifications required to support the formation of multiple endospores or intracellular offspring [13]. For example, *Epulopiscium* sp. type B are intestinal symbionts of the unicornfish *Naso tonganus* and can reach lengths of 200–300 μm and widths of 50–60 μm [11,54]. While the production of endospores has been observed in related morphotypes [12], *Epulopiscium* sp. type B does not produce endospores and does not reproduce by binary fission (Figure 1). Instead, each cell forms two or more intracellular offspring in a process that repeats daily [54-56].

The formation of these offspring has been described in stages that parallel stages of endospore formation [54-56]. Stage 0 mother cells contain large offspring (daughter cells) that show no signs of the initiation of the next generation of offspring (i.e. granddaughter cells). Stage I is defined as offspring cells that have coalesced DNA at the poles. Stage II cells have straight polar septa, but not all polar DNA is inside the newly formed offspring. Stage II-III includes cells with curved polar septa, indicating the start of polar cell engulfment, and all polar DNA has been translocated into the offspring. Stage III cells contain small, fully engulfed offspring, with a length-to-width ratio of less than 2:1 while the offspring in stage IV* cells have a ratio greater than 2:1. After engulfment, the two processes diverge and changes occurring in *Epulopiscium* are not well understood so later stages are not indicated in this model. Offspring continue to grow until they fill the mother-cell cytoplasm. In time, the mother-cell envelope splits open and the offspring are released.

Previous work has shown that the division protein FtsZ localizes to the poles of an *Epulopiscium* cell in a similar way to FtsZ in *B. subtilis* during endospore formation [54]. In *B. subtilis*, both potential division sites appear fully functional and a second, partial polar septum occasionally forms at the forespore-distal pole although the second septum eventually regresses [57,58]. With *Epulopiscium*, rapid sequential or simultaneous bipolar division occurs [54]. Putative homologs for SpoIIE, SpoIIAA, SpoIIAB and σ^F^ also have been identified in the *Epulopiscium* genome [55]. Moreover, the expression pattern of *spoIIE* in *Epulopiscium* during offspring formation [55] is very similar to that seen in sporulating *B. subtilis*[59]. These results suggest that *Epulopiscium* uses cell-specific activation of alternative sigma factors in offspring development.

Here we sequenced and examined the draft genome of *Epulopiscium* sp. type B to determine the extent of conservation of the sporulation genetic program. A list of conserved “core” sporulation genes was assembled and used to determine which of the core sporulation genes are conserved in the *Epulopiscium* genome and its spore-forming relative *Cellulosilyticum lentocellum* DSM 5427. As predicted, we found a number of homologs to genes with sporulation-specific functions in *Epulopiscium* and even more of the core genes conserved in *C. lentocellum*. These results begin to define the genetic mechanisms that may be used for offspring production and development in *Epulopiscium*.

## Results and discussion

### *Epulopiscium* sp. type B draft genome

The draft genome of *Epulopiscium* sp. type B was used to assess the conservation of sporulation gene homologs in this bacterium. *Epulopiscium* sp. type B has become our model for genome studies because *N. tonganus* harbors morphologically and genetically homogenous populations of these *Epulopiscium* cells. Clone libraries of 16S rRNA gene fragments generated from amplified DNA using primers (27F and 1492R) to conserved regions of the gene consistently yield a single phylotype [60,61]. Although the genome was not assembled completely, the project produced approximately 2.7 Mbp of sequence in 92 large contigs ranging from 12 – 119 kb in length. The remaining data, consisting of small contigs less than 12 kb and well-represented single reads (singletons), comprise another 13.7 Mbp. This data set likely represents the *Epulopiscium* genome and may contain some highly repetitive DNA from the fish host or other abundant inhabitants of the *N. tonganus* intestinal tract that may have been carried along during *Epulopiscium* cell isolation.

To determine the completeness of the *Epulopiscium* draft genome, we analyzed the 92 large contigs using two approaches. First, we identified the total number of rRNAs and tRNAs encoded by the genome. We found four 5S, four 23S, and three 16S rRNA genes in addition to 31 tRNAs covering all amino acids except histidine, phenylalanine, and serine. These values are about one-third less the rRNAs and tRNAs encoded by *C. lentocellum* DSM 5427, the closest relative with a complete genome sequence [62]. We also analyzed the small contigs and singletons in the *Epulopiscium* draft genome and found additional functional RNAs. This resulted in a grand total of nine 5S, five 16S and five 23S rRNAs, as well as 66 tRNAs covering all amino acids for the *Epulopiscium* draft genome. These numbers should be viewed with caution as some copies may represent overlapping contigs that did not assemble. Second, we performed a clusters of orthologous genes (COG) analysis using the predicted proteome from the 92 large contigs of the *Epulopiscium* draft genome and compared the percent proteins encoded in each category against a similar analysis of all completely sequenced genomes in either the phylum Firmicutes or the genus *Clostridium* (Table 1). We found that the percentage of proteins in each COG category in the *Epulopiscium* draft genome was comparable to the percentages of either the Firmicutes or the Clostridia. One noteworthy difference was seen in the category of carbohydrate transport and metabolism, which was exceptionally high in *Epulopiscium,* compared to the other Firmicutes groups. Taken together, these data indicate that this draft is somewhat incomplete, although it is difficult to say with absolute certainty the level of completeness.

**Table 1:** **COG analysis of the *Epulopiscium* sp. type B genome**

**COG Category**	**Code**	***Epulopiscium*sp. type B**	***Clostridium***	**Firmicutes**
**Information storage and processing**				
Translation, ribosomal structure and biogenesis	**J**	7.57%	5.91%	6.95%
RNA processing and modification	**A**	0.00%	0.00%	0.00%
Transcription	**K**	5.35%	9.19%	8.05%
Replication, recombination and repair	**L**	4.17%	5.43%	6.20%
Chromatin structure and dynamics	**B**	0.00%	0.03%	0.03%
**Cellular processes and signaling**				
Cell cycle control, cell division, chromosome partitioning	**D**	1.94%	1.24%	1.29%
Nuclear structure	**Y**	0.00%	0.00%	0.00%
Defense mechanisms	**V**	1.60%	2.81%	2.39%
Signal transduction mechanisms	**T**	5.42%	6.92%	4.89%
Cell wall/membrane/envelope biogenesis	**M**	5.49%	5.60%	5.36%
Cell motility	**N**	4.31%	2.73%	1.51%
Cytoskeleton	**Z**	0.00%	0.01%	0.01%
Extracellular structures	**W**	0.00%	0.00%	0.00%
Intracellular trafficking, secretion, and vesicular transport	**U**	1.74%	1.44%	1.53%
Posttranslational modification, protein turnover, chaperones	**O**	3.13%	2.76%	2.99%
**Metabolism**				
Energy production and conversion	**C**	5.97%	5.94%	5.17%
Carbohydrate transport and metabolism	**G**	16.60%	7.26%	8.09%
Amino acid transport and metabolism	**E**	10.56%	8.42%	8.86%
Nucleotide transport and metabolism	**F**	2.78%	2.84%	3.33%
Coenzyme transport and metabolism	**H**	5.00%	4.13%	4.10%
Lipid transport and metabolism	**I**	2.22%	1.97%	2.36%
Inorganic ion transport and metabolism	**P**	5.28%	4.87%	5.22%
Secondary metabolites biosynthesis, transport and catabolism	**Q**	0.49%	1.03%	1.10%
**Poorly characterized**				
General function prediction only	**R**	11.46%	11.09%	11.20%
Function unknown	**S**	6.53%	8.36%	9.39%
Total Genomes Analyzed		1	32	300

For the identification of potential sporulation gene homologs in *Epulopiscium*, contigs and singletons were concatenated into a single pseudomolecule. The position of each junction between adjacent fragments was noted so that any chimeric open reading frames formed during pseudomolecule assembly could be identified.

### The core sporulation gene list

As a starting point, a comprehensive list of sporulation genes was compiled based on previous studies of *B. subtilis* 168 and its derivatives, since this is by far the most thoroughly characterized sporulation program for any member of the Firmicutes. The initial list of 732 genes included those described using classic genetic approaches as well as putative sporulation genes uncovered in transcriptional array analyses of sporulation regulons [29,53,63-65]. BLAST searches refined the list, narrowing it to genes that are conserved in both endospore-forming Bacilli and Clostridia. Further refinement of the list eliminated genes with homologs in non-spore-forming Firmicutes.

Several notable genes that were eliminated are essential in the *B. subtilis* model of sporulation, including the phosphorelay protein Spo0B, and associated histidine kinases, transcription factors RsfA, GerR and GerE, and the intercellular signal transduction proteins SpoIIQ and SpoIVFA. Our inability to identify a SpoIIQ homolog agrees with the results of a previous study [1]. Likewise, the limited distribution of *B. subtilis* phosphorelay homologs among endospore-forming bacteria has been noted previously [66,67] and it is likely that clostridia use a different set of kinases for activation of Spo0A [68,69]. For example, recent genetic studies demonstrated phosphorylation of Spo0A in *C. acetobutylicum* by novel orphan histidine kinases [68,69].

A few key early sporulation proteins (Spo0A, Spo0J, Soj, SpoIIIE and SpoIIIJ), known to function during normal growth in *B. subtilis,* were placed back on the core list. All of these are part of the σ^A^ regulon and were first identified as essential for efficient sporulation. Spo0A determines entry into sporulation. Soj and Spo0J are members of the ParA/B family of partitioning proteins that regulate transcription of early sporulation genes and assist in maintaining chromosome architecture [31]. The FtsK homolog SpoIIIE aids in chromosome separation during binary fission [70], and during sporulation it is responsible for chromosome translocation into the developing forespore [18,19]. The location of genes on the chromosome and timing of translocation affects cell-specific expression of genes, which impacts key molecular events such as the activation of sporulation sigma factors [71-73]. SpoIIIJ is a membrane protein translocase that, in addition to its vegetative growth function, appears to play a key role in the activation of σ^G^ during sporulation [41,74]. In the end, our core list included 147 genes (Table 2).

**Table 2:** **Core sporulation genes identified in the* B. subtilis *168 genome**

**GENE**	**REG**	**FUNCTION**
*aprX*	K	alkaline serine protease
*bofA*	E	inhibitor of the pro-σ^K^ processing machinery
*cotI*	K	spore coat protein
*cotJB*	E	component of the inner spore coat
*cotJC*	E	component of the inner spore coat
*cotS*	K	spore coat protein
*csfB*	F	anti-σ^G^ factor
*cwlD*	E, G	N-acetylmuramoyl-L-alanine amidase/germination
*cwlJ*	E	cell wall hydrolase/germination, cortex lytic
*dacB*	E	D-alanyl-D-alanine carboxypeptidase (penicillin-binding protein 5)
*dacF*	F, G	D-alanyl-D-alanine carboxypeptidase (penicilin-binding protein)
*gerAA*	F, G	component of the germination receptor GerA
*gerAB*	F, G	component of the germination receptor GerA
*gerAC*	F, G	component of the germination receptor GerA
*gerBA*	G	component of germinant receptor B
*gerBB*	G	component of germinant receptor B
*gerBC*	G	lipoprotein component of the germination receptor B
*gerKA*	G	spore germination receptor subunit
*gerKB*	G	spore germination receptor subunit
*gerKC*	G	spore germination receptor subunit
*gpr*	F, G	germination protease
*jag*	A	SpoIIIJ-associated RNA/ssDNA-binding protein
*kamA*	E	lysine 2,3-aminomutase
*kapD*	E,K	inhibitor of the KinA pathway to sporulation
*lonB*	F	ATP-dependent protease/forespore-specific
*mmgC*	E	short chain acyl-CoA dehydrogenase
*ntdA*	E	biosynthesis of neotrehalosadiamine (amino-sugar antibiotic)/aminotransferase
*pbpG*	F, G	penicillin-binding protein (also known as *ywhE*)
*pbpI*	E, F	penicillin-binding protein PBP4B/mother cell specific
*pdaA*	G	exported N-acetylmuramic acid deacetylase/cortex lysis
*prkA*	E	serine protein kinase/not well characterized
*sigE*	A, 0A	sporulation sigma factor/mother cell only
*sigF*	H, 0A	sporulation sigma factor/forespore only
*sigG*	F, G	sporulation sigma factor/forespore only
*sigK*	E, K	sporulation sigma factor/mother cell only
*sleB*	G	spore cortex-lytic enzyme
*soj*	A, 0A	chromosome partitioning protein/transcriptional regulator/negative regulation of sporulation initiation
*splB*	G	spore photoproduct (thymine dimer) lyase
*spmA*	E	spore maturation protein/spore dehydration
*spmB*	E	spore maturation protein/spore dehydration
*spo0A*	A, H, 0A	two-component response regulator central for the initiation of sporulation/"master regulator"
*spo0F*	H, 0A	two-component response regulator involved pathway leading to phosphorylation of Spo0A
*spo0J*	A	site-specific DNA-binding protein/chromosome positioning near the pole and transport through the polar septum/antagonist of Soj-dependent inhibition of sporulation initiation
*spoIIAA*	H, 0A	anti-anti-sigma factor (antagonist of SpoIIAB)
*spoIIAB*	H, 0A	anti-σ^F^ factor
*spoIID*	E	autolysin required for complete dissolution of the sporulation septum
*spoIIE*	A, 0A	serine phosphatase (σ^F^ activation)/polar septum formation
*spoIIGA*	A, 0A	protease processing pro-σ^E^
*spoIIIAA*	E	ATP-binding stage III sporulation protein/mother cell signalling for σ^G^ activation
*spoIIIAB*	E	stage III sporulation protein/mother cell signalling for σ^G^ activation
*spoIIIAC*	E	stage III sporulation protein/mother cell signalling for σ^G^ activation
*spoIIIAD*	E	stage III sporulation protein/mother cell signalling for σ^G^ activation
*spoIIIAE*	E	stage III sporulation protein/mother cell signalling for σ^G^ activation
*spoIIIAF*	E	stage III sporulation protein/mother cell signalling for σ^G^ activation
*spoIIIAG*	E	stage III sporulation engulfment assembly protein/mother cell signalling for σ^G^ activation
*spoIIIAH*	E	stage III sporulation ratchet engulfment protein/mother cell signalling for σ^G^ activation
*spoIIID*	E	transcriptional regulator of σ^E^ and σ^K^-dependent genes
*spoIIIE*	A	spore DNA translocase
*spoIIIJ*	A	protein translocase/essential for activation of σ^G^
*spoIIM*	E	autolysin for dissolution of the septal cell wall
*spoIIP*	E, F, G	spore autolysin
*spoIIR*	F	endopeptidase/activation of σ^E^
*spoIVA*	E	morphogenetic protein required for proper spore cortex formation and coat assembly
*spoIVB*	F, G, 0A	regulatory membrane-associated serine protease/intercompartmental signalling of pro-σ^K^ processing and activation in the mother-cell
*spoIVFB*	E	membrane metalloprotease required for the processing of pro-σ^K^
*spoVAA*	G	dipicolinic acid uptake by the developing spore
*spoVAB*	G	dipicolinic acid uptake by the developing spore
*spoVAC*	G	dipicolinic acid uptake by the developing spore
*spoVAD*	G	dipicolinic acid uptake by the developing spore
*spoVAEB*	G	dipicolinic acid uptake by the developing spore
*spoVAF*	G	dipicolinic acid uptake by the developing spore
*spoVB*	E	spore cortex synthesis
*spoVD*	E	penicillin-binding protein
*spoVE*	E	spore cortex peptidoglycan synthesis
*spoVFA*	K	spore dipicolinate synthase subunit A
*spoVFB*	K	spore dipicolinate synthase subunit B
*spoVR*	E	spore cortex synthesis
*spoVS*	H	regulator required for dehydration of the spore core and assembly of the coat
*spoVT*	F, G	transcriptional regulator of σ^G^-dependent genes
*spsF*	K	putative glycosyltransferase/spore coat polysaccharide synthesis
*spsG*	E, K	putative glycosyltransferase/spore coat polysaccharide synthesis
*spsJ*	E, K	putative dTDP-glucose 4,6-dehydratase/spore coat polysaccharide synthesis
*sspA*	G	small acid-soluble spore protein (alpha-type SASP)
*sspB*	G	small acid-soluble spore protein (beta-type SASP)
*sspC*	G	small acid-soluble spore protein (alpha/beta-type SASP)/SPβ phage protein
*sspD*	G	small acid-soluble spore protein (alpha/beta-type SASP)
*sspF*	G	small acid-soluble spore protein (alpha/beta-type SASP)
*tlp*	F, G	small acid-soluble spore protein
*yaaH*	E	spore peptidoglycan hydrolase
*yabG*	K	sporulation-specific protease
*yabP*	E	spore protein involved in the shaping of the spore coat
*yabQ*	E	membrane protein of the forespore/essential for spore cortex
*ybaN*	E	polysaccharide deacetylase involved in sporulation
*ybdM*	G	putative protein kinase
*ycgF*	E	putative amino acid export permease
*ydfS*	K, G	hypothetical protein
*ydhD*	E	spore cortex lytic enzyme
*yerB*	0A	putative lipoprotein
*yfkQ*	G	putative spore germination protein
*yfkR*	G	putative spore germination protein
*yfkT*	G	putative spore germination integral inner membrane protein
*yfnG*	K	putative sugar-phosphate cytidylyltransferase
*yfnH*	K	putative FAD-dependent oxido-reductase
*ygaK*	K	putative FAD-dependent oxido-reductase
*yhbH*	E	hypothetical protein
*yhcB*	G	putative oxidoreductase associated
*yhcV*	G	putative oxidoreductase
*yhfW*	F	putative Rieske [2Fe-2S] oxygenase
*yisY*	E	putative hydrolase
*ykuD*	K	murein transglycosylase
*ykuS*	F	hypothetical protein
*ykvU*	E	spore membrane protein involved in germination
*ylaK*	E	putative phosphate starvation inducible protein
*ylbB*	F	putative oxidoreductase
*ylbJ*	E	putative factor required for spore cortex formation
*ymxH*	E	hypothetical protein
*yndD*	G	putative spore germination protein
*yndE*	G	putative spore germination integral inner membrane protein
*yndF*	G	putative spore germination lipoprotein
*yngE*	E	putative propionyl-CoA carboxylase
*yngI*	E	AMP-binding domain protein
*yngJ*	E	acyl-CoA dehydrogenase, short-chain specific
*yoaR*	G	putative factor for cell wall maintenance or synthesis
*yobN*	E	putative amine oxidase
*ypeB*	G	spore membrane component
*yqfC*	E	hypothetical protein
*yqfD*	E	stage IV sporulation protein
*yqgT*	E	putative gamma-D-glutamyl-L-diamino acid endopeptidase
*yqhO*	E	hypothetical protein
*yraD*	G	putative spore coat protein
*yrbG*	E, G	hypothetical protein
*yrkC*	K	putative dioxygenase; cupin family
*ytcA*	K	putative UDP-glucose dehydrogenase
*ytcC*	K	putative glucosyltransferase
*ytfJ*	F	hypothetical protein
*ytlA*	K	putative ABC transporter component
*ytlC*	K	putative ABC transporter component, ATP-binding
*ytlD*	K	putative permease of ABC transporter
*ytvI*	E	putative permease
*ytxC*	E	hypothetical protein
*yunB*	E, K	putative protein involved in spore formation
*yutH*	F	spore coat-associated protein
*ywjD*	G	putative UV damage endonuclease
*yyaC*	F	hypothetical protein
*yyaE*	E	putative oxidoreductase
*yyaO*	K	hypothetical protein
*yyBI*	E	inner spore coat protein

### An overview of the core sporulation genes found in *Epulopiscium* and *C. lentocellum*

We were concerned that the phylogenetic distance between *B. subtilis* and *Epulopiscium* may bias the distribution of conserved genes we would be able to identify. To gauge how much evolutionary divergence is impacting the recovery of homologs in *Epulopiscium*, we compared our core list of sporulation genes to the *C. lentocellum* genome as well. *Cellulosilyticum lentocellum* DSM 5427 is the closest endospore-forming relative of *Epulopiscium* with an available completely sequenced genome [62]. *Epulopiscium* sp. type B and *C. lentocellum* are members of the family Lachnospiraceae and the two share 91% 16S rRNA sequence identity. Of the 4,185 predicted protein-coding genes in the *C. lentocellum* genome, an *Epulopiscium* sp. type B gene is the top BLAST hit for 546 of these genes (data not shown).

Of the 147 core sporulation genes, we found 87 homologs in the *C. lentocellum* genome and 57 putative homologs in the *Epulopiscium* sp. type B genome (Table 3). All of the sporulation genes found in the *Epulopiscium* genome were also found in the *C. lentocellum* genome. In the list of conserved genes, two code for members of a family of functionally redundant small acid-soluble proteins (SASPs) that bind to and protect spore DNA from damage [4,75]. One of the SASPs is similar to two paralogs of this family from *B. subtilis* and is represented as *sspC/F*. In both *Epulopiscium* and *C. lentocellum*, at least five genes are represented from each of the four sporulation sigma factor regulons, as well as genes in the Spo0A regulon with σ^H^ and σ^A^ promoters. Of the *C. lentocellum* homologs not seen in the *Epulopiscium* genome, 14 code for “y” genes that are expressed during sporulation, although their roles have yet to be characterized. These genes are equally distributed between pre- and post-engulfment regulons, but only two are expressed in the forespore in *B. subtilis*.

**Table 3:** **Genes conserved in* Epulopiscium *and* C. lentocellum***

**GENE**	**PROMOTER(S)**	***Epulopiscium***	***C. lentocellum***
**σ**^**A**^**regulon**			
*jag*			**X**
*sigE*		**X**	**X**
*soj*		**X**	**X**
*spo0A*		**X**	**X**
*spo0J*		**X**	**X**
*spoIIE*		**X**	**X**
*spoIIGA*		**X**	**X**
*spoIIIE*	σ^A^	**X**	**X**
*spoIIIJ*		**X**	**X**
**σ**^**H**^**regulon**			
*sigF*		**X**	**X**
*spoIIAA*		**X**	**X**
*spoIIAB*		**X**	**X**
**σ**^**F**^**regulon**			
*dacF*	σ^G^	**X**	**X**
*gpr*	σ^G^	**X**	**X**
*lonB*		**X**	**X**
*sigG*	σ^F^, σ^G^	**X**	**X**
*spoIIR*		**X**	**X**
*spoIVB*	σ^F^, σ^G^	**X**	**X**
*spoVT*	σ^G^	**X**	**X**
*yhfW*			**X**
*ytfJ*			**X**
*yyaC*		**X**	**X**
**σ**^**E**^**regulon**			
*bofA*			**X**
*cotJB*			**X**
*cotJC*			**X**
*cwlD*	σ^G^	**X**	**X**
*dacB*		**X**	**X**
*kamA*			**X**
*prkA*		**X**	**X**
*sigK*		**X**	**X**
*spmA*		**X**	**X**
*spmB*		**X**	**X**
*spoIID*	σ^E^	**X**	**X**
*spoIIIAA*	σ^E^	**X**	**X**
*spoIIIAB*	σ^E^	**X**	**X**
*spoIIIAC*	σ^E^	**X**	**X**
*spoIIIAD*	σ^E^	**X**	**X**
*spoIIIAE*	σ^E^	**X**	**X**
*spoIIIAF*	σ^E^	**X**	**X**
*spoIIIAG*	σ^E^	**X**	**X**
*spoIIIAH*	σ^E^	**X**	**X**
*spoIIID*	σ^E^	**X**	**X**
*spoIIP*	σ^G^	**X**	**X**
*spoIVA*	σ^E^	**X**	**X**
*spoVB*		**X**	**X**
*spoVD*		**X**	**X**
*spoVE*		**X**	**X**
*spoVR*		**X**	**X**
*spsJ*			**X**
*yaaH*			**X**
*yabP*	σ^E^	**X**	**X**
*yabQ*	σ^E^	**X**	**X**
*yhbH*		**X**	**X**
*ylbJ*			**X**
*yngI*			**X**
*yqfC*	σ^E^	**X**	**X**
*yqfD*	σ^E^	**X**	**X**
*yqgT*			**X**
*yqhO*			**X**
*ytvI*		**X**	**X**
**σ**^**G**^**regulon**			
*gerKA*			**X**
*gerKB*			**X**
*gerKC*			**X**
*pdaA*		**X**	**X**
*splB*			**X**
*spoVAA*			**X**
*spoVAB*			**X**
*spoVAC*			**X**
*spoVAD*			**X**
*spoVAEB*			**X**
*spoVAF*		**X**	**X**
*sspB*		**X**	**X**
*sspC/F*		**X**	**X**
*ybdM*		**X**	**X**
*ydfS*			**X**
**σ**^**K**^**regulon**			
*cotS*		**X**	**X**
*spoVFA*			**X**
*spoVFB*	σ^K^	**X**	**X**
*yabG*		**X**	**X**
*yfnG*		**X**	**X**
*yfnH*		**X**	**X**
*ygaK*			**X**
*yrkC*			**X**
*ytcC*			**X**
*ytlA*			**X**
*ytlC*			**X**
*ytlD*			**X**

### Conservation of the master regulator Spo0A in *Epulopiscium*

**Figure 2:**
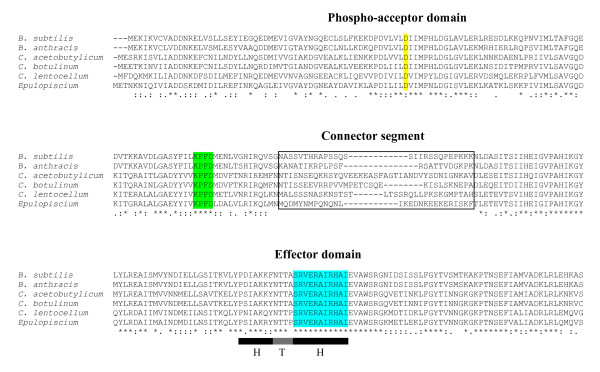
**An alignment of Spo0A homologs.** The predicted amino acid sequences of Spo0A from *B. subtilis* 168, *B. anthracis* Ames, *C. acetobutylicum* ATCC 824, *C. botulinum* ATCC 3502, *Cellulosilyticum lentocellum* DSM 5427 and *Epulopiscium* sp. type B were aligned using CLUSTALΩ. The conserved phosphorylation site (highlighted in yellow), the conformational switch (in green) and the DNA recognition helix (light blue) are found in all homologs. The connector segment (outlined in black) links the upstream phospho-acceptor and downstream effector domains. Shaded bars below the effector domain indicate the helix-turn-helix (HTH) DNA binding motif.

Although clostridia do not have a phosphorelay system homologous to that used by *B. subtilis*, Spo0A is conserved in the genomes of all known endospore-forming bacteria. The *Epulopiscium* sp. type B genome contains an unambiguous Spo0A homolog (Figure 2). The *Epulopiscium* Spo0A homolog is 51% identical to Spo0A of *B. subtilis*. Conserved residues in both phospho-acceptor and DNA binding domains [76] are located in the putative *Epulopiscium* Spo0A homolog as well. This suggests that although offspring production seems hard-wired in *Epulopiscium* and there is no obvious need to adapt the timing of entry into offspring production to environmental conditions, Spo0A is still important for initiating this process or possibly other metabolic transitions. *Bacillus* spp*.* use the phosphorylation state and abundance of Spo0A to engage in activities, such as cannibalism, to maintain a growing population for as long as possible prior to resorting to sporulation [26]. In clostridia, Spo0A also dictates metabolic transitions [77]. For example, in *Clostridium acetobutylicum*, Spo0A activation shifts the cell from acid production during exponential phase growth to solvent production during stationary phase in addition to entry into sporulation [78]. We searched the *Epulopiscium* genome for homologs of the newly discovered *C. acetobutylicum* Spo0A kinases that are important for developmental and metabolic transitions [69], however none of these appear to be conserved in the *Epulopiscium* genome. The future identification and characterization of kinases or phosphatases in *Epulopiscium* that interact with Spo0A may provide information about the environmental and cellular cues that regulate population level developmental or physiological transitions.

### The central regulatory network conserved in endospore-forming bacteria appears conserved in *Epulopiscium*

**Figure 3:**
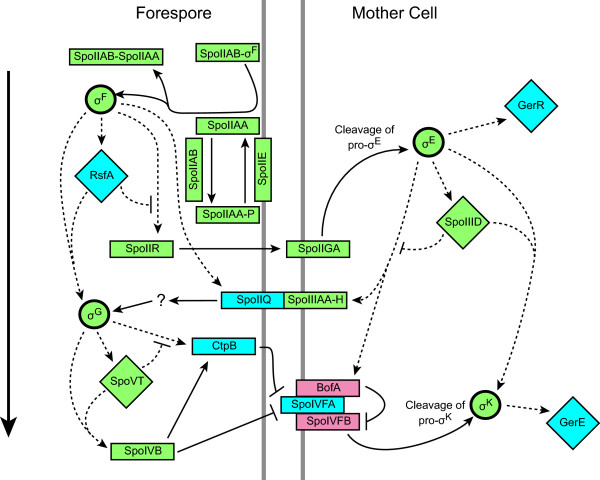
**Conservation of the *B. subtilis* sporulation regulatory cascade in *Epulopiscium* sp. type B.** Sporulation-specific sigma factors (circles), associated transcription factors (diamonds) and other signal transduction or regulatory proteins involved in sigma activation (rectangles) are shown. Colors of the proteins indicate the gene presence in *Epulopiscium* (green), on the core list but not in *Epulopiscium* (red), and absence from the core list (blue). Control of gene expression is indicated by dotted lines and arrows. Signaling pathways and other protein interactions are denoted with solid lines and arrows. Temporal transcriptional progression through the cascade is shown by the position on the diagram with earlier stages near the top. A detailed explanation of the regulatory cascade as it occurs in *B. subtilis* is provided in the text. Figure is adapted from de Hoon *et al*. (2010).

In *B. subtilis*, the sequential activation of cell-specific sigma factors directs compartmentalized gene expression that determines the different fates of the mother cell and forespore [1]. The presence of homologous genes suggests a similar system likely also functions in *Epulopiscium* developmental progression (Figure 3). In a previous publication, we described the σ^F^ homolog of *Epulopiscium* and a structural analysis of the proteins required for σ^F^ activation: SpoIIE, SpoIIAA, and SpoIIAB [55]. In the present study, we uncovered genes coding for all additional sporulation sigma factors (σ^E^, σ^G^ and σ^K^) and homologs for the intercellular signal transduction proteins responsible for the activation of σ^E^ (SpoIIR and SpoIIGA) and σ^G^ (SpoIIIAA-AH and SpoIIIJ). In *B. subtilis*, SpoIIQ interacts with SpoIIIAA-AH in an intracellular signaling system required for the activation of σ^G^[45]. The gene coding for SpoIIQ is absent in clostridia [1] therefore involvement of SpoIIQ is a *Bacillus*-specific innovation that likely does not represent the ancestral mode of cell-cell communication used to trigger late forespore sigma factor activation.

Mechanisms to activate the late mother-cell sigma factor, σ^K^, are apparently the most specialized of the sporulation sigma factors. In *B. subtilis*, the gene encoding σ^K^ contains a 48 kb insertion sequence called the *skin* (sigma K intervening) element that must first be excised from the mother-cell chromosome by the recombinase SpoIVCA [79,80]. The reconstituted gene produces an inactive pro-protein which must be processed by SpoIVFB but BofA and SpoIVFA inhibit SpoIVFB activity [47,48]. These three proteins are transcribed by σ^E^ in the mother cell and localize to the outer forespore membrane. SpoIVB and CtpB, regulated by σ^G^ in the forespore, inhibit the actions of BofA and SpoIVFA allowing cleavage of pro-σ^K^[44,81]. In this way, σ^K^ operates in the mother cell only after σ^G^ has been activated in the forespore. Only homologs for *spoIVB* and *ctpB* were found in *Epulopiscium*. Note that *ctpB* homologs are found in many non-spore-forming bacteria, which is why it is not included in the core list. The gene coding for SpoIVFA is absent in clostridia and although *spoIVFB* and *bofA* met the requirements to be included on the core list, neither is conserved in many clostridia and only *bofA* was found in the *C. lentocellum* genome. The weak conservation of these proteins outside of the Bacilli indicates that the intricate mechanism of σ^K^ activation described in *B. subtilis* is a recent innovation.

In addition to conservation of sporulation sigma factors and many of the proteins responsible for regulating their activation, we found homologs of transcription factors SpoIIID and SpoVT. These proteins work together with their associated sigma factor (σ^E^ and σ^G^, respectively) to modulate transcription of genes downstream in the process. Other transcription factors that function during sporulation in *B. subtilis* (RsfA, GerR and GerE) are not conserved in the clostridia. These feed-forward loops appear to be a Bacilli-specific amendment. Based on these results, we conclude that gene regulation by sporulation-specific sigma factors and their associated transcription factors is highly conserved in *Epulopiscium*. With the exception of *bofA*, all of the core sporulation genes coding for components of the central regulatory cascade that are conserved in the *C. lentocellum* genome are also conserved in *Epulopiscium*.

### Engulfment genes are highly conserved in *Epulopiscium*

SpoIID, SpoIIP and SpoIIM are required for degradation of septal peptidoglycan and progression toward forespore engulfment in *B. subtilis*[21]. We found *Epulopiscium* genes that code for homologs of SpoIID and SpoIIP but could not identify a SpoIIM homolog. SpoIIM appears highly conserved in spore-forming bacteria but is curiously absent from the Lachnospiraceae, as the genome of *C. lentocellum* also lacks an apparent homolog of *spoIIM* (Table 3). The *Epulopiscium spoIIP* initially retrieved from the pseudomolecule did not meet the requirements to be considered a homolog in this analysis, as it appeared to be missing its 5´ end. In *B. subtilisspoIIP* has its own σ^E^ promoter [82] but is also the second gene in an operon with *gpr*[63], and this operon structure is widely conserved in *Bacillus* and *Clostridium* spp. A truncated *gpr* homolog was identified in the *Epulopiscium* draft genome. Based on this information, PCR and sequence analysis was used to recover the complete *Epulopiscium gpr* - *spoIIP* operon.

**Figure 4:**
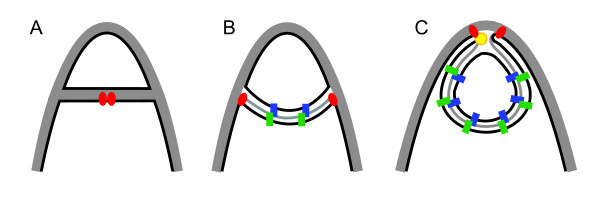
***Epulopiscium* engulfment model.** The *Epulopiscium* genome codes for all of the genes known to be essential for engulfment in *B. subtilis*, except *spoIIM* and *spoIIQ* which are also absent in *C. lentocellum*. **A**) SpoIID and SpoIIP assemble into a complex (red ovals) at the division septum and degrade the septal peptidoglycan. **B**) As the mother-cell membrane wraps around the offspring, the IIDP complex tracks along the leading edge where it is involved in interactions with the mother-cell peptidoglycan and synthesis of offspring cell wall. **C**) When it reaches the cell tip, membrane fusion is mediated by SpoIIIE (yellow circle). During engulfment, SpoIIIAH (green rectangles) produced in the mother cell and a hypothetical protein (blue rectangles) from the offspring cell bind and prevent backward movement of the mother-cell membrane. In this diagram, black lines indicate membranes and grey peptidoglycan.

Other proteins that function during engulfment in *B. subtilis* are SpoIIB, SpoIIIE, SpoIIIAH and SpoIIQ. SpoIIB likely plays a role in the localization of the SpoIIDMP complex in *B. subtilis*[20] but it is not conserved in the clostridia. Null mutants of *spoIIB* in *B. subtilis* are oligosporogenous, and this sporulation defect becomes more pronounced when combined with a *spoVG* mutation [83]. In addition to its DNA translocase activity, SpoIIIE has been implicated in the fusion of the leading edge of the mother-cell membrane after migrating around the forespore [84,85]. We identified homologs of SpoIIIE in both *Epulopiscium* and *C. lentocellum.* Beyond the role of the SpoIIIAH - SpoIIQ complex in σ^G^ activation, these two proteins may perform an important role during engulfment. It has been suggested that as the mother-cell membrane migrates around the forespore, these proteins bind to one another near the leading edge of the mother-cell membrane to prevent it from retreating back toward the midcell [22]. Homologs of the *spoIIIA* operon were identified in *C. lentocellum* and *Epulopiscium*. With the exception of SpoIIM and SpoIIQ, all of the known proteins required for engulfment in *B. subtilis* are coded for in the *Epulopiscium* genome (Figure 4). Neither SpoIIM nor SpoIIQ are coded for in the *C. lentocellum* genome.

### Late stage sporulation proteins in the *Epulopiscium* genome

A small number of late sporulation genes were identified in the *Epulopiscium* sp. type B genome. This may be an underestimate as many of the proteins in this category are very small and functionally redundant. For example, it is possible that some coat proteins have diverged to a degree that they could not to be detected by our methods. Regardless, *B. subtilis* uses more than 75 proteins for cortex and coat formation, spore maturation and germination of which *Epulopiscium* has retained 17 possible homologs. In comparison, the *C. lentocellum* genome has 31. The genes retained in *Epulopiscium* may perform novel functions, or are relics of its spore-forming ancestry. Previous studies have identified sporulation homologs in the genomes of non-sporulating members of the Firmicutes [2]. Additionally, many surgeonfish intestinal symbionts that are close relatives of *Epulopiscium* sp. type B form endospores [12], and would likely need late sporulation proteins similar to those in *B. subtilis* and the clostridia for the formation of cortex and coat as well as spore maturation and germination.

**Figure 5:**
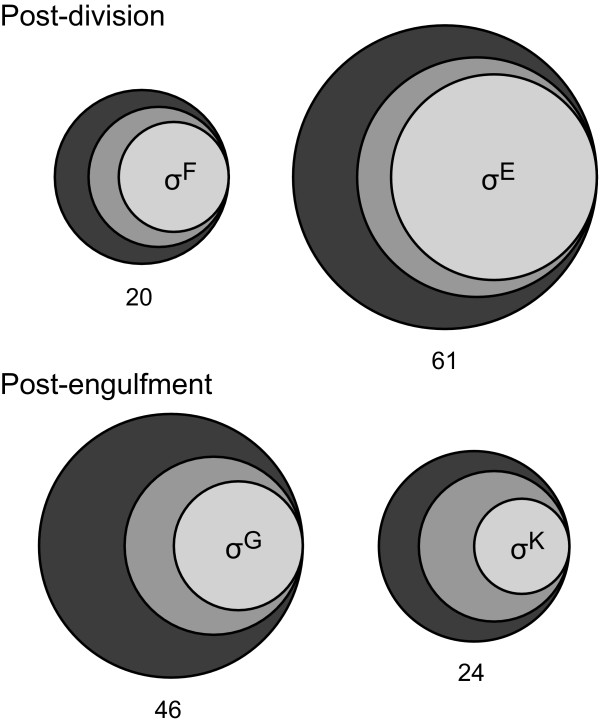
**Distribution of core sporulation genes conserved in the *Epulopiscium* and *C. lentocellum *genomes by regulon.** Venn diagrams represent the conservation of genes in the four sporulation-specific sigma factor regulons. Circle size corresponds to the number genes on the core list for *B. subtilis* (outermost circle), *C. lentocellum* (middle circle) and *Epulopiscium* (inner circle). The numbers below each diagram indicate the total number of genes from *B. subtilis* in each regulon. Some genes were counted more than once if they are members of multiple regulons as indicated in Table 2

Based on their functions in *B. subtilis*, we classified the late sporulation genes located in *C. lentocellum* and/or *Epulopiscium*. With the notable exception of genes coding for SASPs (*sspBsspC/F*) or SASP degradation (*gpr*), genes involved in resistance properties of a mature spore and germination signal receptors (*gerK* operon) are more highly conserved in *C. lentocellum*. Specifically, *C. lentocellum* has the nine genes required for the synthesis and forespore-uptake of dipicolinic acid (DPA) while *Epulopiscium* has only one of these genes. Likewise, only *C. lentocellum* has *splB,* which codes for the DNA repair enzyme spore-photoproduct lyase. Surprisingly, all of the ten genes on the list involved in cortex biosynthesis or other cortex-associated properties that are conserved in *C. lentocellum* are also found in *Epulopiscium*. In addition, *spoIVAyabP* and *yabQ*, which encode scaffolding proteins important for cortex and coat morphogenesis [86-88], were found in both *C. lentocellum* and *Epulopiscium*. We speculate that the synthesis of a cortex-like peptidoglycan may accommodate several *Epulopiscium* characteristics including rapid offspring growth, emergence of offspring from the mother cell or other functions that support a large bacterial cell. Four coat protein genes were found in *C. lentocellum* but only one of these appears conserved in *Epulopiscium*. The biased distribution of functional categories represented in *Epulopiscium* suggests that cortex biosynthetic machinery has been retained, as well as the DNA-protective SASPs, and these proteins may still serve some function. Overall, the pattern of conserved genes in *C. lentocellum* compared with *Epulopiscium* (Figure 5) supports our hypothesis that there is a strong selection to retain early stage sporulation gene homologs in *Epulopiscium* such as those necessary for compartmentalized gene expression, engulfment and developmental progression.

### Promoter analysis of putative developmentally regulated genes

For each of the sporulation genes found in the *Epulopiscium* genome, the region immediately upstream of the predicted start codon (~300 bp) was visually scanned for potential promoter sequences (Table 4). While this analysis is not definitive, we reasoned that identifiable promoters that match their predicted *B. subtilis* counterparts would reinforce the predictions placing genes in particular regulons. Consensus of the −10 and −35 promoter sequences from *B. subtilis* promoters were used [53,63,89]. We were able to identify σ^F^ promoters upstream of *sigG* and *spoIVB,* and σ^E^ promoters upstream of *spoIID*, the *spoIIIA* operon, *spoIIID*, the *yabPQ* operon and the *yqfCD* operon. σ^G^ promoters were found upstream of *dacFcwlDsigGspoIVBspoVT* and the *gpr* – *spoIIP* operon. Only one gene, *spoVFB*, was found to have a σ^K^ promoter. Our analysis of the putative *dacF* homolog indicated that approximately 50% of the 5´ end of the gene was present in the pseudomolecule, however, we were unable to obtain a full-length gene by PCR. The gene *spoVFB* codes for the ß-subunit of DPA synthase and is usually located in a bicistonic operon downstream of *spoVFA*, the gene for the α-subunit of the DPA synthase [90]. DPA is found in abundance only in mature endospores [91] and is a distinct feature of the phase-bright spores of the *Epulopiscium*-like symbionts of *Naso lituratus*[12]. Although we were unable to recover *spoVFA* from *Epulopiscium*, we did find homologs of *spoVFA* and *spoVFB* adjacent to each other in the *C. lentocellum* genome.

**Table 4:** **Promoters found upstream of sporulation gene homologs in the* Epulopiscium *genome**

**GENE/OPERON**	**PROMOTER**							
	**σ**^**F**^		**σ**^**E**^		**σ**^**G**^		**σ**^**K**^	
	**−35**	**−10**	**−35**	**−10**	**−35**	**−10**	**−35**	**−10**
*sigG*	GTATA	GGGTATCCTA			GTATA	TATCCTA		
*spoIVB*	GTATA	GGCAATTTTA			GTATA	AATTTTA		
*spoIID*			TTATAAGT	TCATATAATT				
*spoIIIA*			TAATGATT	GCATATACTG				
*spoIIID*			TAATATAT	GCATATTATT				
*spoIVA*			TCATATCC	ACATATAGTT				
*yabPQ*			GAATAATT	AAATATAAAT				
*yqfCD*			GAATACTT	GCATAATATG				
*dacF*					GTATA	AATAATA		
*cwlD*					GAATT	GATAATA		
*gpr-spoIIP*					GATTA	TATATTA		
*spoVT*					GCATA	CATAATA		
*spoVFB*							TCACA	TCATATTATA
*B. subtilis* consensus^*a*^	**G**TA**TA**	**G**GNA**A**N**A**M**T**R	TYA**T**ATTT	KC**ATA**NAN**T**N	GN**AT**A	C**A**W**A**M**TA**	KC**AC**M	GC**ATA**NNN**T**A

^*a*^ Consensus sequences from *B. subtilis* were based on Wang *et al.* (2006) and Eichenberger *et al.* (2004). Bold indicates a highly conserved base. N = A, T, G, C; R = A, G; Y = C, T; K = T, G; M = A, C; W = A, T.

## Conclusions

The comparative analysis of the draft *Epulopiscium* sp. type B genome with the complete *C. lentocellum* genome substantiates our hypothesis that the production of intracellular offspring in *Epulopiscium* evolved from endospore formation. All of the genes identified in *C. lentocellum* that function in engulfment as well as the core transcriptional regulatory cascade, and the associated intracellular communication network that coordinates sigma factor activation, were found in the *Epulopiscium* genome. While we could identify homologs of late sporulation genes, a large proportion of these were not recovered from *Epulopiscium*. Since we used a draft genome to explore the conservation of sporulation genes in *Epulopiscium*, it is possible that we did not recover all of the sporulation genes retained in this genome, however, we would not expect a functional bias in the distribution of genes recovered. Therefore the fewer late genes recovered in *Epulopiscium* probably reflect the reduction of this class of genes in the evolution of its genome.

Although *Epulopiscium* sp. type B is closely related to surgeonfish intestinal symbionts that form multiple endospores to reproduce [12], it appears that type B cells may no longer have the genetic capacity to form a dormant and fully resistant endospore. Many intestinal anaerobes, from harmful pathogens to benign commensals, use endospores for effective dispersal between vertebrate hosts [8,10,92]. The ability of an intestinal bacterium to produce an endospore should be valuable to survival. Why then would *Epulopiscium* sp. type B lose this trait? We speculate that the perpetuation of large cell size and the ability to maintain a longer residence in an individual host may be factors that contributed to the loss of dormancy and associated resistance traits in offspring development in this lineage. The large size of *Epulopiscium* sp. type B cells may be important to maintain their position in the gut and to avoid predation by the ciliate predators that cohabitate the *N. tonganus* intestinal tract [61]. The growth and development of offspring within a metabolically active mother cell may be essential for maintaining these benefits throughout the life cycle of an individual. It is also possible that the manifestation of some resistance traits may simply be impossible for a cell as large as an *Epulopiscium* sp. type B offspring. For example, the formation of a flawless spore coat may be physically impossible for this size of cell.

In the study outlined here, we generated the first genome sequence for any *Epulopiscium* species and use it to provide insight into the evolution of a novel form of cellular reproduction in the *Epulopiscium* lineage. Based on its phylogenetic position among endospore-forming lineages, we reason that formation of active intracellular offspring in *Epulopiscium* sp. type B is a recent modification of the sporulation program. Given the number of sporulation gene homologs found in the *Epulopiscium* genome, we consider it highly unlikely that the developmental program was assembled through multiple horizontal transfer events. Our comparative study reveals that genes essential for physical (e.g. engulfment of the offspring cell) and regulatory mechanisms (e.g. alternative sigma factors and intracellular communication) have been maintained in a live-offspring-bearing cell. We also found that genes involved in the synthesis of a modified form of peptidoglycan have been conserved. These may be important for offspring growth and intracellular development. Clearly further functional studies are required to test the above models and identify additional mechanisms acquired or modified during evolution of offspring formation in *Epulopiscium*. The findings presented here will provide a framework to begin to assess genetic programs expressed during development in *Epulopiscium*.

## Methods

### *Epulopiscium* sample collection and DNA extraction

*Naso tonganus* were collected by spear on outer reefs in the vicinity of Lizard Island, Great Barrier Reef, Australia. Sections of the gut were removed and the contents were fixed in 80% ethanol. Samples were stored at −20 °C upon arrival at the laboratory.

*Epulopiscium* cells were manually selected from fixed intestinal contents, using a standard Gilson pipettor and a Nikon SMZ-U dissecting microscope. Cell lysis and DNA extraction were performed as previously described [61]. Briefly, *Epulopiscium* type B cells were incubated with 100 μg/ml Proteinase K for one hour at 50 °C. DNA was extracted with phenol:chloroform and precipitated. After rinsing with 70% ethanol, the DNA was resuspended in TE (10 mM Tris, 1 mM EDTA, pH 8) buffer.

### Genome sequencing and analysis

A draft genome sequence was generated using a random shotgun approach and paired-end Sanger sequencing. Sequence reads, providing approximately 8-fold coverage of the estimated 4 Mbp genome were assembled using a combination of the Celera Assembler [93] and TIGR Assembler [94]. To improve assembly, reads were first sorted based on G + C content and only reads that had 23 – 53% G + C were retained. The resulting reads were assembled again and open reading frames (ORFs) predicted using GLIMMER [95]. A BLAST [96] analysis of predicted ORFs against the National Center for Biotechnological Information (NCBI) non-redundant protein (nr) database was used to remove non-bacterial sequences. This strategy yielded 92 large contigs 12 kb to 119 kb in length. These contigs are deposited in GenBank under the accession number NZ_ABEQ01000000.

Draft genome assessment of the 92 large contigs was performed using two approaches. First, the total number of rRNA and tRNA genes were predicted using RNAmmer [97] and tRNAscan-SE [98], respectively. This analysis was also performed on the small contigs and singletons in the *Epulopiscium* draft genome. Second, a clusters of orthologous genes (COG) analysis [99] was performed using the *Epulopiscium* predicted proteome. Each protein was compared against a local COG database obtained from NCBI (ftp://ftp.ncbi.nih.gov/pub/mmdb/cdd/, accessed: 06/23/2011) using RPSBLAST [100] and the total number of proteins in each COG category was tabulated and represented as a percent of the number of COG-annotated proteins in the genome. This analysis was also performed for all sequenced genomes belonging to the phylum Firmicutes and genus *Clostridium* using the complete sequenced microbial genome collection from the NCBI (http://www.ncbi.nlm.nih.gov/genomes/lproks.cgi, accessed: 06/23/2011).

### Generation of a core sporulation gene list

An initial list of sporulation genes was created based on four studies describing the regulons of the four sporulation-specific sigma factors and the transcription factor Spo0A from *B. subtilis*[29,53,63,64]. Other genes reported in the GenoList Comparative Microbial Genome Browser [65] with putative sporulation functions in *B. subtilis* 168 were added to this list. Any gene coding for a protein with a known vegetative growth function was removed. This list was refined further by BLASTP (http://blast.ncbi.nlm.nih.gov/Blast.cgi) searches to determine the distribution of similar proteins in the NCBI nr database. Multiple searches were performed and limited to the phylum Firmicutes, class Clostridia, and if necessary *Clostridium* spp. and *Bacillus* spp. Proteins represented in 15 strains of endospore-forming bacteria in both class Bacilli and class Clostridia with greater than or equal to 70% query coverage and 20% identity were retained on the list. Proteins with top hits for putative homologs in two or more species within the non-spore-forming genera *ListeriaStreptococcusLactobacillusStaphylococcusEnterococcus*, and *Peptostreptococcus*, indicating a vegetative function, were removed. Several key proteins that were first identified as essential to sporulation (Spo0A, Spo0J, Soj, SpoIIIE and SpoIIIJ) were added back to the list.

### Search for core proteins coded for in the *Epulopiscium* and *C. lentocellum* genomes

All contigs and well-represented single reads from the *Epulopiscium* sp. type B draft genome were concatenated to form a pseudomolecule. Coordinates for all junctions between contigs and single reads were entered into an Excel file for easy reference. The pseudomolecule and the complete *C. lentocellum* DSM 5427 genome [62] were searched for homologs of core proteins using TBLASTN and BLASTP, respectively. For each query, the top hits in the *Epulopiscium* or *C. lentocellum* genome were compared to *Bacillus* and *Clostridium* sequences in the NCBI nr database using BLASTP. If reverse-BLAST searches recovered a top hit that was different from the *B. subtilis* core protein used in the initial BLAST analysis, the protein was eliminated from the list. By this method, additional putative homologs with weak hits (below the original cutoff values) were identified. Operon structure was an additional criterion used to identify homologs. Core sporulation protein homologs identified in *C. lentocellum* were then used to search the *Epulopiscium* genome using TBLASTN. Multiple sequence alignments were performed using ClustalΩ [101].

### Confirmation of *dacF*, *gpr-spoIIP*, *spoVT*, *sspC/F*, *ybdM and yyaC*

Primers were developed to amplify sporulation genes identified in the *Epulopiscium* genome that were located in small contigs or singletons or to link genes in operons predicted from synteny in the genomes of other spore formers (Table 5). PCR was performed using standard reaction conditions, and products were cloned into the pCR 2.1 TOPO vector (Invitrogen) following the protocol provided by the manufacturer. Sequences of the clones were determined using Big Dye Terminator chemistry and an Applied Biosystems Automated 3730 DNA analyzer, performed at the Cornell Life Sciences Core Laboratories Center and analyzed with Geneious Pro 5.4.2 (Biomatters). The sequences for the *gpr-spoIIP* operon [accession number JN402987], *dacF* [JN402985], *spoVT* [JN402986], *sspC/F* [JN402984], *ybdM* [JN402982], and *yyaC* [JN402983] are available from GenBank.

**Table 5:** Primers used in this study

**DESIGNATION**	**SEQUENCE (5′ TO 3′)**
GprendF	ATAGACGCATTAGGAGCACG
SpoIIPbegR	GCTTAGCGGACTTTGTATCACC
EpuloGprF	GAGAACATTGGTATTACAGGCG
EpuloGprR	GCTTGCATATATCACCTCCTTG
EpuloSpoIIPF	CATTGCTGTTCACCCAGGTA
EpuloSpoIIP859R	GCAGTAACCTTAGACGCA
EpuloSpoIIP684F	CAAAGTATGGGCTAAATGTATTGC
EpuloSpoIIPR	CAAAACAACAGACATCACCG
EpuloDacFF	GAGCCCCTGATTGTAACATTT
EpuloDacFR1	GGTTAATCCAAATTCACTTTCGCC
EpuloSpoVTF	TTTAGTATTATCAAGAGAAAAACAGCAT
EpuloSpoVTR	TGAACATTTGTCAAGATATAAATGCAA
EpuloSspC/FF	CTCCAAAATAATTTAGGAATATTGTCC
EpuloSspC/FR	TACACAGAAGTACCCCTTTGC
EpuloYbdMF	GAGTGGTTCTTTTAGTGGCTCTG
EpuloYbdMR	AACATGACCTCACACTGGCA
EpuloYyaCF	GCGGTGTTTGTTGTAAGTGC
EpuloYyaCR	TACACCCGAAGAATTAAGCA

## Competing interests

The author(s) declare that they have no competing interests.

## Authors’ contributions

ERA and DAM designed the study and wrote the initial manuscript draft. KDC and ERA collected samples. DAM and GS designed and performed the computational analyses. DAM and ERA interpreted results. All authors contributed to writing the manuscript and approved the final draft.

## References

[B1] de Hoon MJ Eichenberger P Vitkup D Hierarchical evolution of the bacterial sporulation network Curr Biol 2010 20 17 R735 R745 10.1016/j.cub.2010.06.031 20833318 PMC2944226

[B2] Onyenwoke RU Brill JA Farahi K Wiegel J Sporulation genes in members of the low G + C Gram-type-positive phylogenetic branch (Firmicutes) Arch Microbiol 2004 182 2–3 182 192 15340788 10.1007/s00203-004-0696-y

[B3] Nicholson WL Munakata N Horneck G Melosh HJ Setlow P Resistance of Bacillus endospores to extreme terrestrial and extraterrestrial environments Microbiol Mol Biol Rev 2000 64 3 548 572 10.1128/MMBR.64.3.548-572.2000 10974126 PMC99004

[B4] Setlow P I will survive: DNA protection in bacterial spores Trends Microbiol 2007 15 4 172 180 10.1016/j.tim.2007.02.004 17336071

[B5] Angert ER Alternatives to binary fission in bacteria Nat Rev Microbiol 2005 3 3 214 224 10.1038/nrmicro1096 15738949

[B6] Smith LD Clostridium oceanicum, sp. n., a sporeforming anaerobe isolated from marine sediments J Bacteriol 1970 103 3 811 813 4097533 10.1128/jb.103.3.811-813.1970 PMC248161

[B7] Klaasen HL Koopman JP Van den Brink ME Bakker MH Poelma FG Beynen AC Intestinal, segmented, filamentous bacteria in a wide range of vertebrate species Lab Anim 1993 27 2 141 150 10.1258/002367793780810441 8501895

[B8] Chase DG Erlandsen SL Evidence for a complex life cycle and endospore formation in the attached, filamentous, segmented bacterium from murine ileum J Bacteriol 1976 127 1 572 583 931952 10.1128/jb.127.1.572-583.1976 PMC233091

[B9] Robinow CF Observations on the structure of Bacillus spores J Gen Microbiol 1951 5 3 439 457 10.1099/00221287-5-3-439 14873888

[B10] Angert ER Losick RM Propagation by sporulation in the guinea pig symbiont Metabacterium polyspora Proc Natl Acad Sci U S A 1998 95 17 10218 10223 10.1073/pnas.95.17.10218 9707627 PMC21488

[B11] Clements KD Sutton DC Choat JH Occurrence and characteristics of unusual protistan symbionts from surgeonfishes (Acanthuridae) of the Great Barrier Reef, Australia Marine Biol 1989 102 403 412 10.1007/BF00428493

[B12] Flint JF Drzymalski D Montgomery WL Southam G Angert ER Nocturnal production of endospores in natural populations of Epulopiscium-like surgeonfish symbionts J Bacteriol 2005 187 21 7460 7470 10.1128/JB.187.21.7460-7470.2005 16237029 PMC1272977

[B13] Angert ER Brooks AE Pace NR Phylogenetic analysis of Metabacterium polyspora: clues to the evolutionary origin of daughter cell production in Epulopiscium species, the largest bacteria J Bacteriol 1996 178 5 1451 1456 8631724 10.1128/jb.178.5.1451-1456.1996 PMC177821

[B14] Ryter A Etude morphologique de la sporulation de Bacillus subtilis Ann Inst Pasteur 1965 108 40 60 14289982

[B15] Kay D Warren SC Sporulation in Bacillus subtilis. Morphological changes Biochem J 1968 109 5 819 824 4972256 10.1042/bj1090819 PMC1187033

[B16] Fitz-James PC Young IE Gould GW, Hurst A Morphology of sporulation The bacterial spore 1969 Academic, New York, N.Y 39 72

[B17] Bylund JE Haines MA Piggot PJ Higgins ML Axial filament formation in Bacillus subtilis: induction of nucleoids of increasing length after addition of chloramphenicol to exponential-phase cultures approaching stationary phase J Bacteriol 1993 175 7 1886 1890 7681431 10.1128/jb.175.7.1886-1890.1993 PMC204252

[B18] Wu LJ Errington J Bacillus subtilis SpoIIIE protein required for DNA segregation during asymmetric cell division Science 1994 264 5158 572 575 10.1126/science.8160014 8160014

[B19] Bath J Wu LJ Errington J Wang JC Role of Bacillus subtilis SpoIIIE in DNA transport across the mother cell-prespore division septum Science 2000 290 5493 995 997 10.1126/science.290.5493.995 11062134

[B20] Aung S Shum J Abanes-De Mello A Broder DH Fredlund-Gutierrez J Chiba S Pogliano K Dual localization pathways for the engulfment proteins during Bacillus subtilis sporulation Mol Microbiol 2007 65 6 1534 1546 10.1111/j.1365-2958.2007.05887.x 17824930 PMC2885130

[B21] Chastanet A Losick R Engulfment during sporulation in Bacillus subtilis is governed by a multi-protein complex containing tandemly acting autolysins Mol Microbiol 2007 64 1 139 152 10.1111/j.1365-2958.2007.05652.x 17376078

[B22] Broder DH Pogliano K Forespore engulfment mediated by a ratchet-like mechanism Cell 2006 126 5 917 928 10.1016/j.cell.2006.06.053 16959571 PMC3266857

[B23] Ellar DJ Spore specific structures and their function Symp Soc Gen Microbiol 1978 28 295 325

[B24] Henriques AO Moran CP Structure and assembly of the bacterial endospore coat Methods 2000 20 1 95 110 10.1006/meth.1999.0909 10610808

[B25] Burbulys D Trach KA Hoch JA Initiation of sporulation in B. subtilis is controlled by a multicomponent phosphorelay Cell 1991 64 3 545 552 10.1016/0092-8674(91)90238-T 1846779

[B26] Fujita M Losick R Evidence that entry into sporulation in Bacillus subtilis is governed by a gradual increase in the level and activity of the master regulator Spo0A Genes Dev 2005 19 18 2236 2244 10.1101/gad.1335705 16166384 PMC1221893

[B27] Veening JW Hamoen LW Kuipers OP Phosphatases modulate the bistable sporulation gene expression pattern in Bacillus subtilis Mol Microbiol 2005 56 6 1481 1494 10.1111/j.1365-2958.2005.04659.x 15916600

[B28] Lopez D Kolter R Extracellular signals that define distinct and coexisting cell fates in Bacillus subtilis FEMS Microbiol Rev 2010 34 2 134 149 10.1111/j.1574-6976.2009.00199.x 20030732

[B29] Molle V Fujita M Jensen ST Eichenberger P Gonzalez-Pastor JE Liu JS Losick R The Spo0A regulon of Bacillus subtilis Mol Microbiol 2003 50 5 1683 1701 10.1046/j.1365-2958.2003.03818.x 14651647

[B30] Losick R Stragier P Crisscross regulation of cell-type-specific gene expression during development in B. subtilis Nature 1992 355 6361 601 604 10.1038/355601a0 1538747

[B31] Hilbert DW Piggot PJ Compartmentalization of gene expression during Bacillus subtilis spore formation Microbiol Mol Biol Rev 2004 68 2 234 262 10.1128/MMBR.68.2.234-262.2004 15187183 PMC419919

[B32] Gholamhoseinian A Piggot PJ Timing of spoII gene expression relative to septum formation during sporulation of Bacillus subtilis J Bacteriol 1989 171 10 5747 5749 2507532 10.1128/jb.171.10.5747-5749.1989 PMC210425

[B33] Duncan L Losick R SpoIIAB is an anti-sigma factor that binds to and inhibits transcription by regulatory protein σF from Bacillus subtilis Proc Natl Acad Sci U S A 1993 90 6 2325 2329 10.1073/pnas.90.6.2325 8460142 PMC46079

[B34] LaBell TL Trempy JE Haldenwang WG Sporulation-specific sigma factor σ29 of Bacillus subtilis is synthesized from a precursor protein, P31 Proc Natl Acad Sci U S A 1987 84 7 1784 1788 10.1073/pnas.84.7.1784 3104904 PMC304525

[B35] Duncan L Alper S Losick R SpoIIAA governs the release of the cell-type specific transcription factor σF from its anti-sigma factor SpoIIAB J Mol Biol 1996 260 2 147 164 10.1006/jmbi.1996.0389 8764397

[B36] Clarkson J Campbell ID Yudkin MD Physical evidence for the induced release of the Bacillus subtilis transcription factor, σF, from its inhibitory complex J Mol Biol 2004 340 2 203 209 10.1016/j.jmb.2004.04.061 15201047

[B37] Arigoni F Duncan L Alper S Losick R Stragier P SpoIIE governs the phosphorylation state of a protein regulating transcription factor σF during sporulation in Bacillus subtilis Proc Natl Acad Sci U S A 1996 93 8 3238 3242 10.1073/pnas.93.8.3238 8622920 PMC39589

[B38] Karow ML Glaser P Piggot PJ Identification of a gene, spoIIR, that links the activation of σE to the transcriptional activity of σF during sporulation in Bacillus subtilis Proc Natl Acad Sci U S A 1995 92 6 2012 2016 10.1073/pnas.92.6.2012 7892217 PMC42413

[B39] Hofmeister AE Londono-Vallejo A Harry E Stragier P Losick R Extracellular signal protein triggering the proteolytic activation of a developmental transcription factor in B. subtilis Cell 1995 83 2 219 226 10.1016/0092-8674(95)90163-9 7585939

[B40] Jonas RM Weaver EA Kenney TJ Moran CP Haldenwang WG The Bacillus subtilis spoIIG operon encodes both σE and a gene necessary for σE activation J Bacteriol 1988 170 2 507 511 2448286 10.1128/jb.170.2.507-511.1988 PMC210682

[B41] Errington J Appleby L Daniel RA Goodfellow H Partridge SR Yudkin MD Structure and function of the spoIIIJ gene of Bacillus subtilis: a vegetatively expressed gene that is essential for σG activity at an intermediate stage of sporulation J Gen Microbiol 1992 138 12 2609 2618 10.1099/00221287-138-12-2609 1487728

[B42] Partridge SR Errington J The importance of morphological events and intercellular interactions in the regulation of prespore-specific gene expression during sporulation in Bacillus subtilis Mol Microbiol 1993 8 5 945 955 10.1111/j.1365-2958.1993.tb01639.x 8355618

[B43] Camp AH Losick R A novel pathway of intercellular signalling in Bacillus subtilis involves a protein with similarity to a component of type III secretion channels Mol Microbiol 2008 69 2 402 417 10.1111/j.1365-2958.2008.06289.x 18485064 PMC2574792

[B44] Campo N Rudner DZ SpoIVB and CtpB are both forespore signals in the activation of the sporulation transcription factor σK in Bacillus subtilis J Bacteriol 2007 189 16 6021 6027 10.1128/JB.00399-07 17557826 PMC1952037

[B45] Meisner J Wang X Serrano M Henriques AO Moran CP A channel connecting the mother cell and forespore during bacterial endospore formation Proc Natl Acad Sci U S A 2008 105 39 15100 15105 10.1073/pnas.0806301105 18812514 PMC2567499

[B46] Kroos L Kunkel B Losick R Switch protein alters specificity of RNA polymerase containing a compartment-specific sigma factor Science 1989 243 4890 526 529 10.1126/science.2492118 2492118

[B47] Ricca E Cutting S Losick R Characterization of bofA, a gene involved in intercompartmental regulation of pro-σK processing during sporulation in Bacillus subtilis J Bacteriol 1992 174 10 3177 3184 1577688 10.1128/jb.174.10.3177-3184.1992 PMC205984

[B48] Cutting S Roels S Losick R Sporulation operon spoIVF and the characterization of mutations that uncouple mother-cell from forespore gene expression in Bacillus subtilis J Mol Biol 1991 221 4 1237 1256 1942049 10.1016/0022-2836(91)90931-u

[B49] Juan Wu L Errington J Identification and characterization of a new prespore-specific regulatory gene, rsfA, of Bacillus subtilis J Bacteriol 2000 182 2 418 424 10.1128/JB.182.2.418-424.2000 10629188 PMC94291

[B50] Zheng L Halberg R Roels S Ichikawa H Kroos L Losick R Sporulation regulatory protein GerE from Bacillus subtilis binds to and can activate or repress transcription from promoters for mother-cell-specific genes J Mol Biol 1992 226 4 1037 1050 10.1016/0022-2836(92)91051-P 1518043

[B51] Bagyan I Hobot J Cutting S A compartmentalized regulator of developmental gene expression in Bacillus subtilis J Bacteriol 1996 178 15 4500 4507 8755877 10.1128/jb.178.15.4500-4507.1996 PMC178216

[B52] Halberg R Kroos L Sporulation regulatory protein SpoIIID from Bacillus subtilis activates and represses transcription by both mother-cell-specific forms of RNA polymerase J Mol Biol 1994 243 3 425 436 10.1006/jmbi.1994.1670 7966271

[B53] Eichenberger P Fujita M Jensen ST Conlon EM Rudner DZ Wang ST Ferguson C Haga K Sato T Liu JS The program of gene transcription for a single differentiating cell type during sporulation in Bacillus subtilis PLoS Biol 2004 2 10 e328 10.1371/journal.pbio.0020328 15383836 PMC517825

[B54] Angert ER Clements KD Initiation of intracellular offspring in Epulopiscium Mol Microbiol 2004 51 3 827 835 14731282 10.1046/j.1365-2958.2003.03869.x

[B55] Miller DA Choat JH Clements KD Angert ER The spoIIE homolog of Epulopiscium sp. type B is expressed early in intracellular offspring development J Bacteriol 2011 193 10 2642 2646 10.1128/JB.00105-11 21398534 PMC3133141

[B56] Ward RJ Clements KD Choat JH Angert ER Cytology of terminally differentiated Epulopiscium mother cells DNA Cell Biol 2009 28 2 57 64 10.1089/dna.2008.0801 19196050

[B57] Ryter A Schaeffer P Ionesco H Classification cytologique, par leur stade blocage, des mutants de sporulation de Bacillus subtilis Marburg Ann Inst Pasteur 1966 110 305 315 4955547

[B58] Pogliano J Osborne N Sharp MD Abanes-De Mello A Perez A Sun YL Pogliano K A vital stain for studying membrane dynamics in bacteria: a novel mechanism controlling septation during Bacillus subtilis sporulation Mol Microbiol 1999 31 4 1149 1159 10.1046/j.1365-2958.1999.01255.x 10096082 PMC2885269

[B59] Guzman P Westpheling J Youngman P Characterization of the promoter region of the Bacillus subtilisspoIIE operon J Bacteriol 1988 170 4 1598 1609 2832371 10.1128/jb.170.4.1598-1609.1988 PMC211007

[B60] Angert ER Clements KD Pace NR The largest bacterium Nature 1993 362 6417 239 241 10.1038/362239a0 8459849

[B61] Mendell JE Clements KD Choat JH Angert ER Extreme polyploidy in a large bacterium Proc Natl Acad Sci U S A 2008 105 18 6730 6734 10.1073/pnas.0707522105 18445653 PMC2373351

[B62] Miller DA Suen G Bruce D Copeland A Cheng JF Detter C Goodwin LA Han CS Hauser LJ Land ML Complete genome sequence of the cellulose-degrading bacterium Cellulosilyticum lentocellum J Bacteriol 2011 193 9 2357 2358 10.1128/JB.00239-11 21398547 PMC3133088

[B63] Wang ST Setlow B Conlon EM Lyon JL Imamura D Sato T Setlow P Losick R Eichenberger P The forespore line of gene expression in Bacillus subtilis J Mol Biol 2006 358 1 16 37 10.1016/j.jmb.2006.01.059 16497325

[B64] Steil L Serrano M Henriques AO Volker U Genome-wide analysis of temporally regulated and compartment-specific gene expression in sporulating cells of Bacillus subtilis Microbiology 2005 151 Pt 2 399 420 15699190 10.1099/mic.0.27493-0

[B65] Lechat P Hummel L Rousseau S Moszer I GenoList: an integrated environment for comparative analysis of microbial genomes Nucleic Acids Res 2008 36 Database issue D469 D474 18032431 10.1093/nar/gkm1042 PMC2238853

[B66] Stephenson K Hoch JA Evolution of signalling in the sporulation phosphorelay Mol Microbiol 2002 46 2 297 304 10.1046/j.1365-2958.2002.03186.x 12406209

[B67] Stragier P Sonenshein AL, Hoch JA, Losick R A gene odyssey: exploring the genomes of endospore-forming bacteria Bacillus subtilis and its closest relatives 2002 ASM Press, Washington D.C 519 525

[B68] Paredes CJ Alsaker KV Papoutsakis ET A comparative genomic view of clostridial sporulation and physiology Nat Rev Microbiol 2005 3 12 969 978 10.1038/nrmicro1288 16261177

[B69] Steiner E Dago AE Young DI Heap JT Minton NP Hoch JA Young M Multiple orphan histidine kinases interact directly with Spo0A to control the initiation of endospore formation in Clostridium acetobutylicum Mol Microbiol 2011 80 3 641 654 10.1111/j.1365-2958.2011.07608.x 21401736 PMC3097173

[B70] Sharpe ME Errington J Postseptational chromosome partitioning in bacteria Proc Natl Acad Sci U S A 1995 92 19 8630 8634 10.1073/pnas.92.19.8630 7567988 PMC41020

[B71] McBride SM Rubio A Wang L Haldenwang WG Contributions of protein structure and gene position to the compartmentalization of the regulatory proteins σE and SpoIIE in sporulating Bacillus subtilis Mol Microbiol 2005 57 2 434 451 10.1111/j.1365-2958.2005.04712.x 15978076

[B72] Chary VK Xenopoulos P Piggot PJ Blocking chromosome translocation during sporulation of Bacillus subtilis can result in prespore-specific activation of σG that is independent of σE and of engulfment J Bacteriol 2006 188 20 7267 7273 10.1128/JB.00744-06 17015665 PMC1636243

[B73] Eldar A Chary VK Xenopoulos P Fontes ME Loson OC Dworkin J Piggot PJ Elowitz MB Partial penetrance facilitates developmental evolution in bacteria Nature 2009 460 7254 510 514 19578359 10.1038/nature08150 PMC2716064

[B74] Saller MJ Fusetti F Driessen AJ Bacillus subtilis SpoIIIJ and YqjG function in membrane protein biogenesis J Bacteriol 2009 191 21 6749 6757 10.1128/JB.00853-09 19717609 PMC2795313

[B75] Setlow B McGinnis KA Ragkousi K Setlow P Effects of major spore-specific DNA binding proteins on Bacillus subtilis sporulation and spore properties J Bacteriol 2000 182 24 6906 6912 10.1128/JB.182.24.6906-6912.2000 11092849 PMC94814

[B76] Brown DP Ganova-Raeva L Green BD Wilkinson SR Young M Youngman P Characterization of spo0A homologues in diverse Bacillus and Clostridium species identifies a probable DNA-binding domain Mol Microbiol 1994 14 3 411 426 10.1111/j.1365-2958.1994.tb02176.x 7885226

[B77] Dürre P Ancestral sporulation initiation Mol Microbiol 2011 80 3 584 587 10.1111/j.1365-2958.2011.07628.x 21435030

[B78] Ravagnani A Jennert KC Steiner E Grunberg R Jefferies JR Wilkinson SR Young DI Tidswell EC Brown DP Youngman P Spo0A directly controls the switch from acid to solvent production in solvent-forming clostridia Mol Microbiol 2000 37 5 1172 1185 10.1046/j.1365-2958.2000.02071.x 10972834

[B79] Popham DL Stragier P Binding of the Bacillus subtilis spoIVCA product to the recombination sites of the element interrupting the σK-encoding gene Proc Natl Acad Sci U S A 1992 89 13 5991 5995 10.1073/pnas.89.13.5991 1631085 PMC402124

[B80] Stragier P Kunkel B Kroos L Losick R Chromosomal rearrangement generating a composite gene for a developmental transcription factor Science 1989 243 4890 507 512 10.1126/science.2536191 2536191

[B81] Cutting S Driks A Schmidt R Kunkel B Losick R Forespore-specific transcription of a gene in the signal transduction pathway that governs Pro-σK processing in Bacillus subtilis Genes Dev 1991 5 3 456 466 10.1101/gad.5.3.456 1900494

[B82] Frandsen N Stragier P Identification and characterization of the Bacillus subtilis spoIIP locus J Bacteriol 1995 177 3 716 722 7836306 10.1128/jb.177.3.716-722.1995 PMC176648

[B83] Margolis PS Driks A Losick R Sporulation gene spoIIB from Bacillus subtilis J Bacteriol 1993 175 2 528 540 8419299 10.1128/jb.175.2.528-540.1993 PMC196168

[B84] Liu NJ Dutton RJ Pogliano K Evidence that the SpoIIIE DNA translocase participates in membrane fusion during cytokinesis and engulfment Mol Microbiol 2006 59 4 1097 1113 10.1111/j.1365-2958.2005.05004.x 16430687 PMC2885140

[B85] Sharp MD Pogliano K The membrane domain of SpoIIIE is required for membrane fusion during Bacillus subtilis sporulation J Bacteriol 2003 185 6 2005 2008 10.1128/JB.185.6.2005-2008.2003 12618465 PMC150122

[B86] Asai K Takamatsu H Iwano M Kodama T Watabe K Ogasawara N The Bacillus subtilis yabQ gene is essential for formation of the spore cortex Microbiology 2001 147 Pt 4 919 927 11283287 10.1099/00221287-147-4-919

[B87] van Ooij C Eichenberger P Losick R Dynamic patterns of subcellular protein localization during spore coat morphogenesis in Bacillus subtilis J Bacteriol 2004 186 14 4441 4448 10.1128/JB.186.14.4441-4448.2004 15231775 PMC438564

[B88] Piggot PJ Coote JG Genetic aspects of bacterial endospore formation Bacteriol Rev 1976 40 4 908 962 12736 10.1128/br.40.4.908-962.1976 PMC413989

[B89] Amaya E Khvorova A Piggot PJ Analysis of promoter recognition in vivo directed by σF of Bacillus subtilis by using random-sequence oligonucleotides J Bacteriol 2001 183 12 3623 3630 10.1128/JB.183.12.3623-3630.2001 11371526 PMC95239

[B90] Daniel RA Errington J Cloning, DNA sequence, functional analysis and transcriptional regulation of the genes encoding dipicolinic acid synthetase required for sporulation in Bacillus subtilis J Mol Biol 1993 232 2 468 483 10.1006/jmbi.1993.1403 8345520

[B91] Errington J Bacillus subtilis sporulation: regulation of gene expression and control of morphogenesis Microbiol Rev 1993 57 1 1 33 8464402 10.1128/mr.57.1.1-33.1993 PMC372899

[B92] Alvarez Z Abel-Santos E Potential use of inhibitors of bacteria spore germination in the prophylactic treatment of anthrax and Clostridium difficile-associated disease Expert Rev Anti Infect Ther 2007 5 5 783 792 10.1586/14787210.5.5.783 17914913

[B93] Myers EW Sutton GG Delcher AL Dew IM Fasulo DP Flanigan MJ Kravitz SA Mobarry CM Reinert KH Remington KA A whole-genome assembly of Drosophila Science 2000 287 5461 2196 2204 10.1126/science.287.5461.2196 10731133

[B94] Sutton GG White O Adams MD Kerlavage AR TIGR Assembler: A new tool for assembling large shotgun sequencing projects Genome Sequencing Tech 1995 1 9 19 10.1089/gst.1995.1.9

[B95] Delcher AL Bratke KA Powers EC Salzberg SL Identifying bacterial genes and endosymbiont DNA with Glimmer Bioinformatics 2007 23 6 673 679 10.1093/bioinformatics/btm009 17237039 PMC2387122

[B96] Altschul SF Madden TL Schaffer AA Zhang J Zhang Z Miller W Lipman DJ Gapped BLAST and PSI-BLAST: a new generation of protein database search programs Nucleic Acids Res 1997 25 17 3389 3402 10.1093/nar/25.17.3389 9254694 PMC146917

[B97] Lagesen K Hallin P Rodland EA Staerfeldt HH Rognes T Ussery DW RNAmmer: consistent and rapid annotation of ribosomal RNA genes Nucleic Acids Res 2007 35 9 3100 3108 10.1093/nar/gkm160 17452365 PMC1888812

[B98] Lowe TM Eddy SR tRNAscan-SE: a program for improved detection of transfer RNA genes in genomic sequence Nucleic Acids Res 1997 25 5 955 964 9023104 10.1093/nar/25.5.955 PMC146525

[B99] Tatusov RL Galperin MY Natale DA Koonin EV The COG database: a tool for genome-scale analysis of protein functions and evolution Nucleic Acids Res 2000 28 1 33 36 10.1093/nar/28.1.33 10592175 PMC102395

[B100] Marchler-Bauer A Anderson JB Derbyshire MK DeWeese-Scott C Gonzales NR Gwadz M Hao L He S Hurwitz DI Jackson JD CDD: a conserved domain database for interactive domain family analysis Nucleic Acids Res 2007 35 Database issue D237 D240 17135202 10.1093/nar/gkl951 PMC1751546

[B101] Sievers F Wilm A Dineen D Gibson TJ Karplus K Li W Lopez R McWilliam H Remmert M Soding J Fast, scalable generation of high-quality protein multiple sequence alignments using Clustal Omega Mol Syst Biol 2011 7 539 21988835 10.1038/msb.2011.75 PMC3261699

